# Does the Integration of Telehealth into Occupational Therapy Practice Impact Clinical Outcomes for Hand and Upper Limb Rehabilitation? A Matched Case Control Study

**DOI:** 10.5195/ijt.2022.6505

**Published:** 2022-12-13

**Authors:** Kristie J. Harper, Siân Fitzgerald, Png Xiyin, Jordan Kuzich, Soon Hui Leow, Jacques Angela, Courtenay Harris

**Affiliations:** 1 Occupational Therapy Department, Sir Charles Gairdner Hospital, Perth, Western Australia, Australia; 2 Curtin University, School of Allied Health, Perth, Western Australia, Australia; 3 Enable Institute for Health Research, Perth, Western Australia, Australia; 4 Occupational Therapy Department, Rockingham Hospital, Rockingham, Western Australia, Australia; 5 Institute for Health Research, The University of Notre Dame, Perth, Western Australia, Australia; 6 Department of Research, Sir Charles Gairdner Hospital, Perth, Western Australia, Australia

**Keywords:** Hand and upper limb rehabilitation, Occupational therapy, Telehealth, Telemedicine

## Abstract

Health services are capitalizing on the rise of telehealth and seeking to develop sustainable models incorporating telehealth into standard care. Further research is required to explore the service and clinical outcomes of telehealth in occupational therapy hand and upper limb practice. This research utilized a case-control study to explore the feasibility and clinical outcomes of case matched patients who received a telehealth hybrid model versus traditional in-person care. One hundred and two patients were recruited (n=51 in the controls and cases) with a mean age of 45 years. Telehealth was not inferior to standard care with no significant increase in therapy time (p=0.441) or length of referral (p=0.047). There was no difference in clinical adverse events (p=0.741). Patients who received telehealth had significantly less withdrawals from the service (p = 0.031). Patient and therapist satisfaction were high, supporting the ongoing use and continued implementation of telehealth in occupational therapy.

Upper limb injuries such as fractures, dislocations, and soft tissue injuries are common, accounting for approximately 50% of all injuries to the human body (Worboys et al., 2018). Depending on the severity and nature of the hand condition, management varies from conservative (e.g., use of an orthosis and task modification) through to surgical management with post-operative therapy (Worboys et al., 2018). Therapists implement a variety of treatment techniques to improve range of motion, dexterity, and hand use in daily activity, with manual techniques, scar management, and oedema control (Keller et al., 2016; Sloane et al., 2021). The centrality of therapeutic touch to hand therapy lends itself to in-person encounters, close proximity, and direct contact (Sloane et al., 2021). In response to the COVID-19 pandemic, hand and upper clinics restricted in-person appointments to orthoses fabrication, relying on telehealth to support follow up care and rehabilitation (Gajarawala & Pelkowski, 2021; Hagge et al., 2020; Hollander & Carr, 2020; Monaghesh & Hajizadeh, 2020; Pelly et al., 2020). Evidence for the efficacy of telehealth in hand therapy continues to emerge (Cottrell & Russell, 2020; Grona et al., 2018).

Cottrell et al. (2018) performed a systematic review suggesting telehealth delivery of guideline-based recommended care for musculoskeletal conditions (including persistent pain and osteoarthritis) demonstrated similar efficacy to in-person care (Cottrell & Russell, 2020). Telehealth is broadly accepted by consumers, with greater than 80% of primary care clinicians reporting satisfaction with service quality, however questions remain regarding perceived treatment outcomes for hand and upper limb rehabilitation (Acharya & Rai, 2016; Lawford et al., 2018; Sloane et al., 2021). Other studies have identified challenges with the use of telehealth, such as the perceived or real difficulty in developing an effective therapeutic relationship between the clinician and patient and the ability to appropriately diagnose or manage certain conditions (Cottrell & Russell, 2020; Dorsey & Topol, 2016; Harst et al., 2019; Turolla et al., 2020). Other identified barriers include reduced patient engagement and trust regarding telehealth and decreased access to required resources, such as appropriate internet access and teleconferencing equipment (Cottrell & Russell, 2020; Malliaras et al., 2022; Turolla et al., 2020). One study reported that 42% of clinicians working in Australia agreed that telehealth was as effective as in-person care, however only one in four agreed that patients valued telehealth to the same extent (Malliaras et al., 2022). Despite high rates of adoption and reported confidence among clinicians, it is suggested that many clinicians adopted telehealth due to necessity, making it questionable whether they would persist with its use once barriers to in-person care were removed.

However, telehealth does offer growing advantages for people to access care, increasing patient convenience by reducing travel burden (time and costs) (Cottrell et al., 2012; Cottrell et al., 2018; Cottrell & Russell, 2020; Turolla et al., 2020), improved efficiency and creation of flexible work arrangements for clinicians. As such governments are aiming to continue to integrate telehealth to ensure it is a regularly utilised mode of outpatient service delivery (Western Australian Government Department of Health, 2019). Further research is required to explore the service and clinical impact of telehealth in occupational therapy hand and upper limb practice to support future sustainable use. As such, the aim of this study was to integrate telehealth into standard practice through the use of a hybrid model of care and evaluate the feasibility and impact on patient clinical outcomes. The hybrid model of care consisted of an initial in-person session followed by telehealth for ongoing management. This was compared to patients who received traditional in-person care only. It was hypothesized that a hybrid model of telehealth would be feasible, supported by patients and deliver similar, non-inferior clinical outcomes.

## Methods

### Study Design

This research utilized a case-control study to explore the feasibility and clinical outcomes of case matched patients who received a telehealth hybrid model of care versus traditional in-person care. Reporting adhered to the STROBE statement for observational studies (von Elm et al., 2007).

Ethical approval was granted by Curtin University Human Research Ethics Office (HRE2021-0085) and by the Sir Charles Gairdner Osborne Park Health Care Group (QA39167). All patient data was de-identified and analysed in aggregate form to protect participants' privacy and maintain confidentiality.

### Setting and Timeframes

This research was completed at a metropolitan tertiary-level hospital outpatient clinic setting from 2019–2021. Retrospective data was collected for the control group from patient medical records and using Allied Health System (which clinicians use to record daily statistics on patients seen). Control group patients were case-matched by age, gender and diagnosis and received intervention between 2019 and 2020. They were provided with in-person care and did not receive assessment or treatment via telehealth.

Telehealth commenced in 2020 to support COVID-19 restrictions and case group patients were prospectively recruited throughout 2021. Outcomes were collected throughout patients' referral and intervention period and ceased upon their discharge from the hand and upper limb clinic.

### Participants

The inclusion criteria consisted of patients aged 18 years and older referred to the occupational therapy hand and upper limb clinic. All eligible participants had sustained either a traumatic or nontraumatic hand injury which required intervention. Patients were cognitively intact and able to consent to participate in the study. Participants were not required to have any knowledge or skills associated with computers or telehealth technology to participate.

Patients were excluded if they received less than two treatment sessions for both groups. Additionally, patients in the control group were excluded if they received more than one telephone call to avoid introducing any bias into the study design. Rural patients were also excluded due to additional intervention provided by rural occupational therapists.

### Control Group

The control group received traditional in-person occupational therapy care for their upper limb injury. Appointments ranged from 30 minutes to one hour and consisted of assessment, orthosis fabrication, education, and treatments such as oedema management, scar management and upper limb rehabilitation programs. The treatment regime developed and provided did not incorporate any telehealth sessions.

### Intervention

Cases received a tailored program which included an initial in-person consultation and scheduled telehealth sessions for rehabilitation and follow up. Additional in-person appointments were scheduled, if necessary, to address issues such as orthosis adjustment. Telehealth was provided via the telephone or using video conferencing equipment, which was dependent on the patient's diagnosis, individual care needs, patient preference and access to equipment to support telehealth.

### Outcome Measures

An audit tool was developed using REDCap and piloted by three researchers to support data collection and management (Harris et al., 2009; Harris et al., 2019). Study data were collected and managed using REDCap electronic data capture tools hosted by the Western Australia Department of Health. All researchers audited at least five patient files together using the audit tool at two time periods to ensure consistency and inter-rater reliability. Patient descriptive data recorded included primary diagnosis, reason for referral, age, and gender. Feasibility measures consisted of patient's access to telehealth equipment (yes/no), number of sessions completed via telehealth, length of referral (consisting of time from initial referral to patient discharge in weeks) and occupational therapy service events (total number of sessions and therapy time in minutes). Patients' engagement in service provision and attendance rates were measured through early withdrawal from therapy and incidents of ‘did not attends’ (DNA's) for scheduled appointments.

A senior occupational therapist not involved in therapy provision reviewed each patient's clinical outcomes. The senior therapist determined if a patient had been discharged with a good clinical outcome defined as the achievement of therapy goals upon discharge from the hand therapy clinic, without the need for further follow-up referrals. The clinic has a Criteria Lead Discharge system in place, where occupational therapists can directly discharge patients on behalf of medical specialists if the patient meets set discharge criteria, declined the need for further outpatient consultation, injury was not related to workers compensation and a satisfactory outcome was achieved. As such a good clinical outcome was defined as any patient who did not need to be referred for further plastics or orthopaedics review due to complications and this was a binary (yes/no) response. Additionally, the number of adverse events were recorded and included any incidents such as tendon ruptures, stiffness, and pressure injuries from orthosis, etc. where patients were likely to require an extension of therapy. Data for the control group was retrospectively extracted from the patient's medical records and the case data was prospectively recorded. Three researchers, not involved in the patient's care, supported data extraction.

Patient feedback was captured using the Telehealth Usability Questionnaire (Parmanto et al., 2016). The TUQ measures usability of a telehealth system and has established reliability and validity (Parmanto et al., 2016). The questionnaire consists of six categories: usefulness, ease, interface quality, interaction quality, reliability and satisfaction, with patients providing a rating from: 1 (strongly disagree) to 7 (strongly agree) on a Likert scale (Parmanto et al., 2016). There are 21 items with a maximum score of 147. Usefulness referred to the patient's perception of how the telehealth system functions to provide a healthcare interaction/service like the traditional in-person encounter. Interface quality measured the interaction between the patient and the telehealth technology or computer system (Parmanto et al., 2016). This included the quality of the graphical user interface, the ease of navigation, and an overall impression of how the patient interacts with the telehealth system. Reliability referred to how easily the user can recover from an error and how the system provides guidance to the user in the event of error. Satisfaction was related to the patient's overall satisfaction with the telehealth system and how willing the user would be to use the system in the future (Parmanto et al., 2016). Patients were able to provide comments at the end of the questionnaire. This was completed with patients at discharge from the hand and upper limb clinic.

Additionally, therapists provided feedback regarding the implementation of telehealth, if it was successful and how it could be improved. Therapists used a Likert scale (1 = poor to 7 = very good) to indicate their level of engagement with patients via telehealth and their ability to address all aspects of patient care via telehealth.

### Statistical Analysis

Summary descriptors included means, medians and interquartile ranges for continuous data and frequency distributions for categorical data. To test for significant differences, data was analysed using Mann-Whitney *U* test for non-parametric data and Spearman's rho to examine the association between TUQ results and patient's age. The data were analysed using the SPSS program version 27 (IBM Corp, 2017). All hypotheses were 2-sided and p-values <0.05 were considered statistically significant.

## Results

One hundred and two patients were recruited (n=51 in the controls and cases) from the occupational therapy hand and upper limb clinic. Table 1 highlights the demographic and clinical characteristics of the recruited population. The overall study population had a mean age of 45 years (ranging 17–86) and comprised of 54.9% (n=56) male patients. Out of 102 patients, 57.8% (n=59) presented with hand and upper limb fractures; 17.6% (n=18) with tendon rupture or repair; 15.7% (n=16) with dislocations and the remaining 8.9% (n=9) comprised of other hand and upper limb disorders such as tendinitis, carpal tunnel syndrome, Dupuytren contracture, etc. Orthosis fabrication was required for 87% (n=89) of patients. There were no significant baseline differences between the two groups (Table 1).

**Table 1:** Baseline Demographic and Clinical Characteristics of Participants

		All *n=102*	Control *n=51*	Cases *n=51*	p-value
		n (%)	n (%)	n (%)	
**Sex**	Male	56 (54.9)	29 (56.9)	27 (52.9)	0.691
	Female	46 (45.1)	22 (43.1)	24 (47.1)	
					
**Age**	*Mean (SD)*	45.0 (18.2)	44.9 (18.0)	45.1 (18.5)	0.957
					
**Diagnosis**	Distal phalanx fracture	10 (9.8)	5 (9.8)	5 (9.8)	1.000
	Middle phalanx fracture	7 (6.9)	4 (7.8)	3 (5.9)	
	Proximal phalanx fracture	13 (12.7)	6 (11.8)	7 (13.7)	
	Metacarpal fracture	9 (8.8)	5 (9.8)	4 (7.8)	
	Wrist and forearm fracture	20 (19.6)	10 (19.6)	10 (19.6)	
	Elbow fractures/dislocations	2 (2.0)	1 (2.0)	1 (2.0)	
	Digit/thumb dislocation or ligament injury	14 (13.7)	7 (13.7)	7 (13.7)	
	Zone 1–4 extensor tendon injury	7 (6.9)	4 (7.8)	3 (5.9)	
	Zone 5–8 and all thumb zoned extensor tendon injury	6 (5.9)	3 (5.9)	3 (5.9)	
	Flexor tendon injury	1 (1.0)	0 (0.0)	1 (2.0)	
	Zone 2 flexor tenosynovitis	2 (2.0)	1 (2.0)	1 (2.0)	
	Wrist ligament injury	2 (2.0)	1 (2.0)	1 (2.0)	
	Dupuytrens	4 (3.9)	2 (3.9)	2 (3.9)	
		n (%)	n (%)	n (%)	
	Digit based amputations	2 (2.0)	1 (2.0)	1 (2.0)	
	Tumour or ganglion	1 (1.0)	0 (0.0)	1 (2.0)	
	Carpal tunnel syndrome	2 (2.0)	1 (2.0)	1 (2.0)	

### Feasibility

Ninety percent of case patients (n=46) had the correct equipment to engage in telehealth with 76% (n=39) reporting familiarity with utilizing technology. Twenty-two percent (n=11) engaged in telehealth via a tabletop computer, 29% (n=15) using a laptop, 8% (n=4) with a tablet and 41% (n=21) via a smart phone. Eighty-two percent of patients (n=42) were able to set up and utilize telehealth without carer assistance.

Seventy-five percent (n=38) received telehealth with video support. The case group received a median of three in-person sessions supported with two telehealth sessions (Table 2). These patients received a median of 165 minutes of therapy time. In comparison, the control group received a median of five in-person therapy session (p=0.012), resulting in a median of 190 minutes in total therapy time (p=0.441). Length of referral was a median of 10.9 weeks in the case group compared to 8.1 weeks in the control group (p=0.047).

Patients in the control group had higher rates of DNA's with 10 in the control group compared to five in the case group, although this was not statistically significant (p=0.338). However, there was a significant difference between the groups regarding treatment withdrawal (*p* = 0.031), with seven patients withdrawing from the control group compared to zero in the case group.

**Table 2:** Clinical Intervention Provided and Outcomes for Case and Control Group Patients

		All, n (%) *n-102*	Control, n (%) *n=51*	Cases, n (%) *n=51*	p-value
**Intervention**	Orthosis fabrication	89 (87.3)	43 (84.3)	46 (90.2)	0.373
	Manual range of movement	77 (75.5)	46 (90.2)	31 (60.8)	<0.001
	Functional rehabilitation	64 (62.7)	21 (41.2)	43 (84.3)	<0.001
	Scar and oedema management	29 (28.4)	13 (25.5)	16 (31.4)	0.510
	Sensory retraining	8 (7.8)	4 (7.8)	4 (7.8)	1.000
**Overall number of in-person sessions**	*Median (Interquartile Range) [Min-Max]*	4 (3, 6)	5 (4, 7)	3 (3, 6)	0.012
		[1–18]	[1–18]	[1–10]	
**In-person sessions**	1	7 (6.9)	2 (3.9)	5 (9.8)	0.141
	2–5	59 (57.8)	27 (52.9)	32 (62.7)	
	6–10	33 (32.4)	19 (37.3)	14 (27.5)	
	>10	3 (2.9)	3 (5.9)	0 (0.0)	
**Good clinical outcome**	No	18 (17.6)	12 (23.5)	6 (11.8)	0.119
	Yes	84 (82.4)	39 (76.5)	45 (88.2)	
**Adverse events**	No	92 (90.2)	47 (92.2)	45 (88.2)	0.741
	Yes	10 (9.8)	4 (7.8)	6 (11.8)	
**Number of ‘Did not attends’**	0	78 (76.5)	37 (72.5)	41 (80.4)	0.338
	1	15 (14.7)	10 (19.6)	5 (9.8)	
	2	8 (7.8)	3 (5.9)	5 (9.8)	
	3	1 (1.0)	1 (2.0)	0 (0.0)	
**Therapy time (mins)**	*Median (Interquartile Range) [Min-Max]*	172 (110, 265)	190 (120, 290)	165 (110, 240)	0.441
		[30–595]	[30–595]	[38–375]	
**Length of referral**	*Median (Interquartile Range) [Min-Max]*	9.6 (6.0, 14.0)	8.1 (5.9, 12.7)	10.9 (6.6, 15.6)	0.047
		[0–32.3]	[0–24.1]	[1.3–32.3]	

### Clinical Outcomes

Eighty-eight percent (n=45) of patients were discharged with a satisfactory clinical outcome in the case group compared to 76.5% (n=39) in the control group (p=0.119) (Table 2). There were six adverse events (i.e., tendon rupture, finger contracture, etc.) reported in the case group compared with four in the control group (p=0.741). As anticipated, higher rates of therapist guided range of movement were recorded in the control group (n=46, 90.2 %) compared to the case group (n=31, 60.8%, p=<0.001). Conversely higher rates of functional rehabilitation were utilized in the case group (n=43, 84.3%) compared to the control group (n=21, 41.2%, p=<0.001).

### Patient and Therapist Perspectives

The TUQ provided information on usability of the telehealth system from a patient perspective (n=51) (Table 3). Patients in the case group had a median score of 136/147 indicating strong levels of satisfaction with the use of telehealth (20/21 for usefulness, 20/21 for ease, 27/28 for interface quality, 27/28 for interaction quality, 17/21 for reliability, and 27/28 for satisfaction). Additional qualitative feedback provided from 19 patients highlighted patients found telehealth provided convenience, reduced travel and was easy to use. One patient reported “*I am so pleased with this system and the way it has enabled me to access therapy at home*.”

However, patients also noted that in-person intervention was required and appreciated initially, but telehealth could support follow-up care, as per “*I feel strongly that telehealth cannot replace in-person therapy especially in the early stages*” and “*I feel telehealth is appropriate in later stages of therapy once splinting is no longer required.*”

Three patients (16%) reported having technical issues accessing telehealth, which affected their ability to communicate with the hand therapist. One patient reported “*feeling disappointed due to not having access to the camera on the computer, which affected their ability to access telehealth*.” Whilst another stated that telehealth enhanced their communication with the therapist, and that the “*instructions were clear*.”

**Table 3:** Telehealth Usability Questionnaire (TUQ) Results for the Case Group (n=51)

Item	Median	Interquartile Range *[Minimum-Maximum]*
1. Telehealth improves my access to occupational therapy services.	7	7, 7 [4–7]
2. Telehealth saves me time by not traveling to the hospital.	7	7, 7 [7–7]
3. Telehealth has met my healthcare occupational therapy needs.	7	7, 7 [7–7]
**Usefulness Scale Summary** *(Items 1–3, maximum score = 21)*	**21**	**20, 21 [17–21]**
4. It was simple to use this system.	7	6, 7 [4–7]
5. It was easy to learn this system.	7	7, 7 [4–7]
6. I believe I could become productive quickly using this system	7	6, 7 [4–7]
**Ease of Use and Learnability Scale Summary** *(Items 4–6, maximum score = 21)*	**21**	**19, 21 [14–21]**
7. The way I interact with this system is pleasant.	7	7, 7 [5–7]
8. I like using this system.	7	6, 7 [4–7]
9. The system is simple and easy to understand.	7	7, 7 [5–7]
10. This system is able to do everything I would want it to be able to do.	7	6, 7 [4–7]
**Interface Quality** *(Items 7–10, Maximum Score = 28)*	**27.5**	**25.75, 28 [23–28]**
11.1 can easily talk to my occupational therapists using the telehealth system.	7	7, 7 [4–7]
12. I can hear my occupational therapist clearly using the telehealth system.	7	7, 7 [4–7]
13. I felt I was able to express myself effectively.	7	7, 7 [6–7]
14. Using the telehealth system, I can see my occupational therapist as well as if we met in person.	7	6, 7 [3–7]
**Interaction Quality** *(Items 11–14, Maximum Score = 28)*	**28**	**18, 28 [26–28]**
15. I think the sessions provided over telehealth are the same as in-person sessions.	5	3, 7 [1–7]
16. Whenever I made a mistake using the system, I could recover easily and quickly.	7	6, 7 [4–7]
17. The system gave error messages that clearly told me how to fix problems.	7	7, 7 [1–7]
**Reliability** *(Items 15–17, Maximum Score =21)*	**19**	**17, 21 [8–21]**
18. I feel comfortable communicating with my occupational therapist using the telehealth system.	7	7, 7 [5–7]
19. Telehealth is an acceptable way to receive occupational therapy.	7	6, 7 [3–7]
20. I would use telehealth again.	7	7, 7 [5–7]
21. Overall, I am satisfied with the telehealth system.	7	7, 7 [4–7]
**Satisfaction** *(Items 18–21, Maximum Score = 28)*	**28**	**26, 28 [19–28]**

The TUQ results were similar for younger and older populations (Table 4). Weak correlation between the TUQ results and patients age (r=0.077, p=0.644) was identified, indicating that satisfaction with telehealth was not dependent on a patient's age. Additionally, satisfaction levels were not influenced by the number of in-person sessions a patient received (r=-0.198, p=0.233).

**Table 4:** Telehealth Usability Questionnaire (TUQ) Results Stratified by Age (Above and Below 60 Years of Age)

		Cases (n=51) *Median (Interquartile Range) [Min-Max]*	p-value
**Usefulness**			
*(Items 1–3, Maximum Score = 21)*	<60	21 (20, 21) [17–21]	0.314
	>60	21 (21, 21) [20–21]	
**Ease of Use and Learnability**			
*(Items 4–6, Maximum Score = 21)*	<60	21 (19, 21) [16–21]	0.350
	>60	20 (17, 21) [14–21]	
**Interface Quality**			
*(Items 7–10, Maximum Score = 28)*	<60	27 (26, 28) [23–28]	0.515
	>60	28 (26, 28) [24–28]	
**Interaction Quality**			
*(Items 11–14, Maximum Score = 28)*	<60	28 (26, 28) [18–28]	0.250
	>60	28 (27, 28) [25–28]	
**Reliability**			
*(Items 15–17, Maximum Score = 21)*	<60	19 (17, 20) [11–21]	0.235
	>60	21 (18, 21) [8–21]	
**Satisfaction**			
*(Items 18–21, Maximum Score = 28)*	<60	28 (26, 28) [19–28]	0.586
	>60	28 (27, 28) [24–28]	

On a Likert scale from 1–7, five therapists provided an average rating of 6/7 for their level of engagement with patients via telehealth and 5/7 for their ability to address all aspects of patient care via telehealth. Reported challenges included an inability to assess joint stiffness or objectively measure strength and range of movement and provide hands-on feedback regarding exercises. Minimal other technical difficulties were reported including telephone call/video dropouts. Therapists felt that adequate technical support was available.

## Discussion

This study has provided evidence regarding the integration of telehealth to treat hand and upper limb injuries in occupational therapy. Patients and therapists were satisfied with a hybrid model of service consisting of in-person and telehealth hand therapy which is likely to be sustainable in future clinical practice. Patients received a median of three in-person appointments supplemented with two telehealth sessions. This approach did not impact the achievement of clinical goals or increase the number of adverse events, which is a finding supported in other studies (Wright-Chisem & Trehan, 2021), indicating that excellent care may be preserved with telehealth use.

The TUQ also highlighted that telehealth was well supported by patients. Previous telehealth feasibility studies employing the TUQ found ratings of five out of seven or higher in programs were feasible (Faett et al., 2013; Parmanto et al., 2013; Serwe et al., 2017). In this study the median rating was above six in all areas. Patients highly rated the usefulness, ease of use, interface quality, reliability, and satisfaction subscales. The lowest scoring subscale was related to interaction quality where patients provided a score of 5/7 for ‘I think the sessions provided over telehealth are the same as in-person sessions’ indicating that in-person care may be perceived to be of higher quality by patients. However overall our study results are in-line with other research findings again confirming that patients were very satisfied with telehealth (Wright-Chisem & Trehan, 2021).

This was also reflected in patient appointment attendance and withdrawals from treatment. No patients withdrew from service provision in the case group compared to seven in the control group. There were also less DNA's in the case group. The option of telehealth may have supported patients to continue participation within therapy.

The same number of therapy appointments were received by both patients in the case and control groups, demonstrating that similar conditions could be managed without an increase in resources using a hybrid approach. Overall, the case group was observed to receive less therapy time, although not significant, spread over a longer referral period. This has implications for service resources and costs. Other studies have reported decreased costs associated with the use of telehealth (Tadley et al., 2021) which could be explored in future research.

Therapists were generally satisfied with the application of telehealth; however, they also highlighted common barriers identified in the literature such as the lack of ability to physically examine the patient (Tadley et al., 2021), the need to increase the use of functional activities to support range of movement exercises during telehealth, and patient anxiety, especially in the earlier stages of management. However, therapists reported success with patients regaining functional use of their upper limb in daily activities, despite complex injuries and therapist's inability to use manual techniques in their rehabilitation. This has also been found in other research by Donelan et al. (2019) and Slone et al. (2021) where positive clinical outcomes were attributed to the quality and efficiency of telehealth care. Therapists were also able to recognise the benefits of telehealth including convenience for patients and less DNA's. However, they were supportive of an initial in-person appointment to establish rapport.

As highlighted in other studies, the use of telehealth necessitated more digital connectivity between therapists and patients to make appointments, e-mail instructions, and send through home programs (Sloane et al., 2021). Initial establishment of the software and equipment was required. Ninety percent of the patients had the right equipment to engage in telehealth highlighting the ease of access. However, in this study 18% of patients still required support to engage with telehealth, which is a consideration regarding resources and therapists' time.

This research has provided insight into a hybrid model of care incorporating telehealth in hand therapy. This study has outlined suitable diagnoses where telehealth could be utilized to deliver similar care.

## Limitations

This was a non-randomized case matched study with several limitations including control data collected retrospectively. However, steps were taken to reduce bias such as developing and piloting an audit tool and having three external research assistants support the data extraction. This study was completed at a single site and therefore the results may not be generalizable to other settings.

Patients were case matched based on three main determinants in health consisting of age, gender, and diagnoses. Although these factors aided in providing appropriate case matches, there are other factors that could be considered in future research to improve the validity of the data such as smoking status, diabetes, and other relevant comorbidities (Bykowski et al., 2011).

## Conclusion

A hybrid model of care incorporating telehealth in a hand and upper limb occupational therapy clinic was not inferior to in-person standard care. Clinical outcomes were maintained, and patients appeared more engaged in therapy with improved attendance and significantly less withdrawals from the service. Overall patients and therapists were satisfied with the service model explored, which may support the ongoing use and continued implementation of telehealth.
